# Unique Organization of Actin Cytoskeleton in Magnocellular Vasopressin Neurons in Normal Conditions and in Response to Salt-Loading

**DOI:** 10.1523/ENEURO.0351-19.2020

**Published:** 2020-04-07

**Authors:** Zsuzsanna Barad, Suleima Jacob-Tomas, Alberto Sobrero, Graham Lean, Amirah-Iman Hicks, Jieyi Yang, Katrina Y. Choe, Masha Prager-Khoutorsky

**Affiliations:** 1 Department of Physiology, McGill University, Montreal, Quebec H3G 1Y6, Canada; 2 Semel Institute for Neuroscience and Human Behavior, David Geffen School of Medicine, University of California, Los Angeles, Los Angeles, CA 90095

**Keywords:** actin, hypothalamus, neuroendocrinology, plasticity, vasopressin, water balance

## Abstract

Magnocellular neurosecretory cells (MNCs) are intrinsically osmosensitive and can be activated by increases in blood osmolality, triggering the release of antidiuretic hormone vasopressin (VP) to promote water retention. Hence, the activity of magnocellular VP neurons is one of the key elements contributing to the regulation of body fluid homeostasis in healthy organisms. Chronic exposure to high dietary salt leads to excessive activation of VP neurons, thereby elevating levels of circulating VP, which can cause increases in blood pressure contributing to salt-dependent hypertension. However, the molecular basis underlying high-salt diet-induced hyperactivation of magnocellular VP neurons remains not fully understood. Previous studies suggest that magnocellular neurosecretory neurons contain a subcortical layer of actin filaments and pharmacological stabilization of this actin network potentiates osmotically-induced activation of magnocellular neurons. Using super-resolution imaging *in situ*, we investigated the organization of the actin cytoskeleton in rat MNCs under normal physiological conditions and after a chronic increase in blood osmolality following 7 d of salt-loading (SL). We found that, in addition to the subcortical layer of actin filaments, magnocellular VP neurons are endowed with a unique network of cytoplasmic actin filaments throughout their somata. Moreover, we revealed that the density of both subcortical and cytoplasmic actin networks in magnocellular VP neurons is dramatically increased following SL. These results suggest that increased osmo-responsiveness of VP neurons following chronic exposure to high dietary salt may be mediated by the modulation of unique actin networks in magnocellular VP neurons, possibly contributing to elevated blood pressure in this condition.

## Significance Statement

Hypothalamic magnocellular neurons secrete antidiuretic hormone vasopressin (VP) into the circulation, promoting vasoconstriction and renal water retention. Regulation of VP secretion is a key factor controlling body fluid homeostasis, and excessive VP secretion contributes to fluid balance disorders such as salt-sensitive hypertension. Using super-resolution analysis of different areas of the rat brain, we show that VP neurons feature a unique actin cytoskeleton comprising a subcortical actin layer and an array of cytoplasmic comet-like structures, which are not present in any other neuronal cell type. Moreover, the density of these unique actin structures is increased in a rodent model of salt-sensitive hypertension, and our findings suggest that this modification may contribute to excessive activation of VP neurons in this model.

## Introduction

Magnocellular neurosecretory cells (MNCs) are located in the hypothalamic supraoptic (SON) and paraventricular (PVN) nuclei, and in smaller numbers within accessory neurosecretory nuclei (Voisin and Bourque, 2002; Gottlieb et al., 2006; Bourque, 2008; Tasker et al., 2017). MNCs project their axons to the posterior pituitary as well as to other areas in the brain and spinal cord where they have neuromodulatory functions (Landgraf and Neumann, 2004; Tasker et al., 2017). The activity of MNCs is modulated in response to changes in plasma osmolality (a measure of total solute concentration in the blood plasma). Increases in plasma osmolality enhance the action potential firing rate of MNCs, stimulating the secretion of vasopressin (VP) from nerve terminals located in the posterior pituitary into the blood circulation. VP is antidiuretic hormone causing water reabsorption by the kidney to restore plasma osmolality. Under normal physiological conditions, even fluctuations as small as 1% in plasma osmolality (e.g., following evaporative water loss during breathing, sweating, and the ingestion of solutes or water) induce an appropriate VP release to maintain body fluid and hemodynamic homeostasis (Verney, 1947; Bourque, 2008).

Increases in circulating VP induced by high salt intake (Ludwig et al., 1996) can cause vasoconstriction via the VP receptor 1 located on smooth muscle cells (Henderson and Byron, 2007). Thus, the increase in VP associated with chronic high-salt diet can lead to water retention and vasoconstriction, factors which can both contribute to elevated blood pressure. Hence, exaggerated activation of VP neurons and increased VP release have emerged as significant factors that might contribute to the pathogenesis of salt-sensitive hypertension (Prager-Khoutorsky et al., 2017). Consistent with this, studies in rats suggest that high-sodium intake leads to a two- to three-fold increase in plasma VP (Ludwig et al., 1996; Danziger and Zeidel, 2015) and VP levels are elevated in other animal models of salt-induced hypertension (e.g., DOCA-salt, one kidney renal wrap; Crofton et al., 1979; DiPette et al., 1982; Hinojosa et al., 1986; Rust and Ekmekcioglu, 2017). Studies in rat models of salt-dependent hypertension have demonstrated that interruption of neurotransmission in osmoregulatory circuits controlling VP release reduces or even normalizes blood pressure (Nakata et al., 1989; Ito et al., 2003; Simmonds et al., 2014). Furthermore, consumption of high amounts of dietary salt leads to hyperactivation of VP neurons and excessive VP secretion (Kim et al., 2011, 2013), contributing to the increase in blood pressure (Kim et al., 2013; Choe et al., 2015; Prager-Khoutorsky et al., 2017). However, the mechanisms by which high dietary salt promotes hyperactivation of VP neurons and excessive secretion of VP, contributing to increased blood pressure, remain incompletely understood (Oh et al., 2016).

Extrinsic factors such as local glial cells (Hussy et al., 1997; Deleuze et al., 1998; Choe et al., 2012), as well as synaptic inputs from neurons located in other osmoregulatory nuclei play an important role in the regulation of VP MNC activity in the SON (Bourque et al., 1994; Armstrong et al., 1996). Further, modulation of inhibitory inputs from arterial baroreceptors (Kim et al., 2013; Choe et al., 2015; Prager-Khoutorsky et al., 2017) in response to chronic high salt intake contributes to the VP-dependent increase in blood pressure in rat models of salt-dependent hypertension.

In addition to these extrinsic mechanisms, magnocellular VP neurons are intrinsically osmosensitive (Mason, 1980; Oliet and Bourque, 1992; Prager-Khoutorsky and Bourque, 2015; Prager-Khoutorsky, 2017). Electrical activity of acutely-isolated MNCs is increased by hypertonicity and inhibited by hypotonicity in the absence of neighboring glial cells and synaptic contacts (Oliet and Bourque, 1992). Intrinsic osmosensitivity of MNCs is dependent on the presence of unique cytoskeletal scaffolds comprising a somatic network of microtubules and a subcortical layer of actin filaments (Prager-Khoutorsky and Bourque, 2015; Prager-Khoutorsky, 2017). Recent studies demonstrated that acutely-isolated MNCs from rat SON contain a thin layer of actin filaments located underneath the plasma membrane (Zhang et al., 2007; Zhang and Bourque, 2008). These studies showed that actin cytoskeleton is involved in the activation of MNCs in response to hypertonicity, as disruption of actin filaments with cytochalasin D inhibits the osmotic activation of acutely isolated SON neurons. Notably, stabilizing the actin network with jasplakinolide increases the osmoresponsiveness of acutely isolated MNCs (Zhang et al., 2007; Prager-Khoutorsky and Bourque, 2010). Similar effects can be achieved by treating MNCs with angiotensin II, an excitatory neuropeptide that can be released from the subfornical organ to enhance VP secretion in conditions associated with abnormal fluid balance (Akaishi et al., 1980; Sgro et al., 1984; Jhamandas et al., 1989). Bath application of angiotensin II on acutely isolated MNCs increases subcortical actin density and potentiates their osmotic activation (Zhang and Bourque, 2008). These findings suggest that modulation of the actin cytoskeleton can underlie increases in intrinsic osmosensitivity of MNCs. However, whether changes in actin cytoskeleton occur in physiological or pathological conditions associated with hyperactivation of VP MNCs and enhanced VP release is not known. Thus, we tested the hypothesis that actin density in VP MNCs is increased in response to chronic exposure of animals to high dietary salt.

## Materials and Methods

### Animals

Male Wistar rats (300–400 g; Charles River Laboratories Inc.) were used throughout this study and treated in strict accordance with the guidelines outlined by the Canadian Council on Animal Care (http://www.ccac.ca/) and experiments adhered to protocols #AUP7948 approved by the Facility Animal Care Committee of McGill University. All animals were kept under a standard 12/12 h light/dark cycle and individually housed in behavioral cages (PhenoMaster, TSE Systems). Food and water were provided *ad libitum*. SL-treated rats were provided with 2% NaCl solution as drinking fluid instead of water for 7 d as described previously (Choe et al., 2015). Animals were habituated to single housing in behavioral cages for at least one week before the beginning of SL, and their water and food intake were monitored throughout the habituation and SL period.

### Immunohistochemistry on tissue sections

Rats were transcardially perfused with 10 ml of PBS and then with 250 ml of PBS containing 4% paraformaldehyde and 0.5% glutaraldehyde, pH adjusted to 6.8 at 37°C. Brains were postfixed for at least 48 h. Coronal sections (50 μm thick) obtained using a vibratome were treated for 15 min in 0.1% NaBH_4_ in cytoskeletal buffer containing the following: 130 mM NaCl, 10 mM MES, 5 mM EGTA, 5 mM MgCl_2_, and 5 mM MgCl_2_, pH adjusted to 6.3. After 1-h incubation with 10% normal goat serum in PBS containing 0.3% Triton X-100, sections were washed and incubated overnight at 4°C with primary antibodies diluted in PBS containing 0.3% Triton X-100. Following washes with PBS, sections were incubated for 2 h with fluorescently-labeled secondary antibodies (1:500), mounted in ProLong Gold Antifade reagent (Life Technologies), and imaged using LSM 880 with AiryScan (Zeiss) or 3-D structured illumination microscopy (3-D SIM) using a DeltaVision OMX V_4_ blaze (Applied Precision, GE Healthcare, Life Sciences).

**Figure 1.**
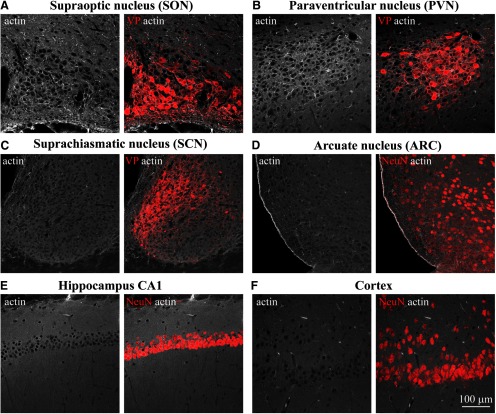
Examination of actin organization in different areas of the rat brain. Confocal imaging of immunostaining for β-actin (white) and VP (red, ***A–C***) or neuronal marker NeuN (red, ***D–F***) analyzed in adult rat brain sections containing SON (***A***), PVN (***B***), SCN (***C***), ARC (***D***), hippocampus CA1 (***E***), cortex (CX; ***F***), and other brain areas (Extended Data Fig. 1-1).

To identify magnocellular and parvocellular PVN neurons, we used anatomic landmarks to distinguish various subdivisions of the PVN (Lechan and Toni, 2000). We only used sections containing mid-PVN level to allow for a more accurate identification of magnocellular and parvocellular divisions, and an additional consideration was given to the size of the somata (Lechan and Toni, 2000). Putative magnocellular VP neurons were analyzed from a region located strictly on lateral wings of the PVN (e.g. Fig. 1*B*); and putative parvocellular VP neurons analyzed were located in the periventricular parvocellular division (a region adjacent to the third ventricle; e.g. Extended Data Fig. 1-1*B*, Lechan and Toni, 2000).

10.1523/ENEURO.0351-19.2020.f1-1 Extended Data Figure 1-1 Examination of actin organization in different brain areas. Confocal imaging of immunostaining for β-actin (white) and VP (red, ***A***, ***B***) or neuronal marker NeuN (red, ***C***, ***D***) analyzed in adult rat brain sections containing accessory nucleus harboring magnocellular VP neurons (***A***), parvocellular division of the PVN (***B***), and hippocampal CA3 (***C***) and DG (***D***). Download Figure 1-1, TIF file.

### Antibodies

β-Actin mouse monoclonal antibodies (catalog #A5441 clone AC-15 or catalog #A5316 clone AC-74, Sigma, 1:500), or β-actin rabbit monoclonal antibody (catalog #4970, Cell Signaling, 1:100), VP guinea pig polyclonal antibody (catalog #403004, SYSY, 1:500), NeuN guinea pig polyclonal antibody (catalog #ABN90, EMD Millipore; 1:500), and VP rabbit polyclonal antibody (VA4, 1:1000; provided by Hal Gainer, NIH) were used in this study. Secondary antibodies were fluorescently-labeled Alexa Fluor-conjugated IgG (488, 568, and 647 nm; 1:500, Invitrogen).

### Confocal microscopy with AiryScan

Confocal microscopy was done using LSM 880 using Apo 20×/0.8 objective (Zeiss), and AiryScan imaging was conducted using Zeiss 63×/1.40 Oil DIC f/ELYRA objective and the AiryScan super-resolution (SR) module with 32-channel hexagonal array GaAsP detector for LSM (Zeiss) and Argon multiline, 651- and 633-nm lasers. The point spread functions (PSFs) for each wavelength were measured using 100-nm fluorescent beads. Based on these PSFs, the maximal resolution of our AiryScan system at the wavelength used for actin imaging (excitation of 488 nm) was 126 nm in the lateral (*xy*) dimension and 346 nm in the axial (*z*) dimension. The acquisition was set to Spot acquisition with 2.46 μs per time point, bit depth was set at eight bits, gain at 750, the pinhole aperture was set to max, and stacks of 20–30 optical sections (170-nm step) were acquired consecutively in different channels (488, 568, and 647 nm). AiryScan super-resolution image stacks were reconstructed using ZEN Black software (Zeiss). Images were analyzed using ZEN Blue software (Zeiss) and FIJI (NIH).

### 3-D SIM

3-D SIM images of fixed samples were acquired using an Applied Precision V4 Blaze OMX system equipped with a 100×/1.4 NA U-PLANAPO objective (Olympus Canada) and two Evolve EM-CCD cameras (Photometrics). Immersion oil was optimized for imaging brain tissue at 488 nm (actin labeling). The PSFs for each wavelength were measured using 100-nm fluorescent beads to assess the resolution. The maximal resolution of our 3-D SIM system at the 488-nm excitation wavelength used for actin imaging was 112 nm in the lateral (*xy*) dimension and 300 nm in the axial (*z*) dimension. Stacks of 48–60 optical sections (125-nm step) were acquired consecutively in three channels (488, 568, and 647 nm) using DeltaVision software (Applied Precision). 3-D super-resolution image stacks were reconstructed using SoftWorx 6.0 using channel specific OTFs and Wiener filter settings of 0.001. The images presented are maximal projections of z-stacks consisting of four consecutive images representing fluorescence collected through 0.5 μm of the tissue or cell (4 × 125 nm).

### Actin cytoskeleton analysis

For all analyses, cells were sampled on both sides of bilateral brain structures from every rat [seven control euhydrated (EU) and eight salt-loaded rats]. Images were collected as z-stacks and for image analysis, individual AiryScan optical sections were used. The analysis might underestimate the length of actin filaments as they can extend through multiple optical sections. To evaluate the subcortical actin layer, cell contours were determined by superimposing fluorescence of neuronal markers (NeuN or VP) with actin images. Line scan profiles were then generated by placing a perpendicular 4-μm-long line across the cell perimeter. Subcortical actin intensity was obtained by subtracting an average intensity 1–2 μm away from the cell perimeter from the average value at the cell perimeter. Subcortical actin layer thickness was calculated from line profiles as distance around cell perimeter at which fluorescence intensity drops to 50%. Three-line profiles per cell were averaged to calculate subcortical actin thickness in each cell. Lines spanning the perimeters of two neighboring neurons were excluded from the analysis.

The density of comet-like actin structures was quantified by counting the number of actin filaments in a 4 × 4 μm window positioned over a central region of the cytoplasm. For cells that have multiple cytoplasmic structures, the length of eight filaments per cell were measured in a 4 × 4 μm cytoplasmic window. The size of the window was expanded until a total of 8 actin filaments were detected. For cells with low numbers of cytoplasmic actin structures, if eight filaments cannot be identified, smaller number of filaments was used to calculate the average length of comet-like structures per cell. Cells that have no cytoplasmic actin structures were excluded from the analysis.

### Statistical analysis

All analyses were performed using Prism 5 (GraphPad Software). Results are reported as mean ± SEM. Data were assessed for normality (Kolmogorov–Smirnov test) and equality of variances (Bartlett’s test or Brown–Forsythe test). If the raw data did not satisfy these conditions, a non-parametric method was used. Data of two groups were compared by a two-tailed, unpaired, parametric Student’s *t* test. For parametric multiple comparisons, we used one-way ANOVA followed by between-group comparisons using Tukey’s *post hoc* test, or a two-way ANOVA followed by between-group multiple comparison Bonferroni’s *post hoc* test. Kruskal–Wallis with *post hoc* Dunn’s test was used for non-parametric multiple comparisons analysis. Additional statistical parameters are shown as extended data figures corresponding to Figures 3, 5, 6 (Extended Data Figs. 3-2, 5-2, and 6-4). The significance level was set at *p* < 0.05; *p < 0.05, **p < 0.01, ***p < 0.001, ****p < 0.0001.

## Results

### Actin organization in magnocellular VP neurons

**Figure 2.**
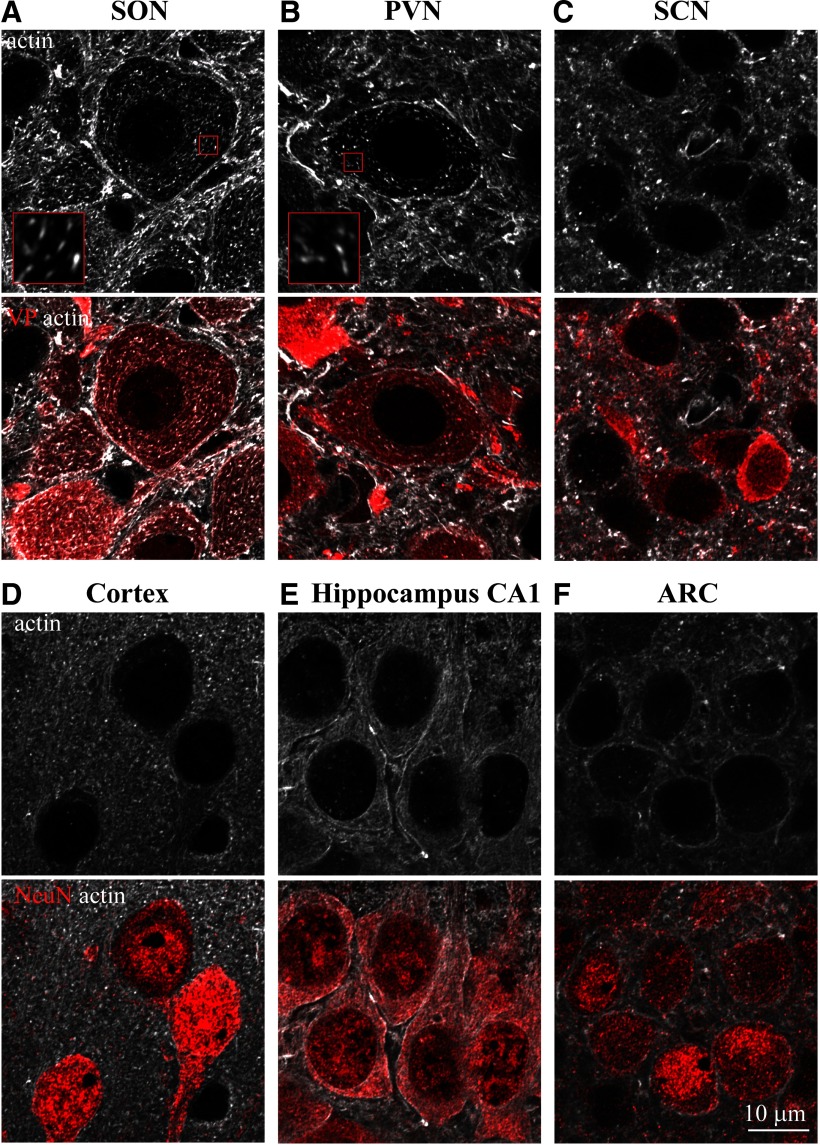
Comparison of actin networks in magnocellular VP neurons and neurons from other brain areas. Immunostaining for β-actin (white) and VP (red, ***A–C***) or neuronal marker NeuN (red, ***D–F***) in adult rat brain sections imaged by confocal microscopy with AiryScan, reveals actin organization in different types of neurons: magnocellular VP neurons from the SON (***A***) and PVN (***B***), SCN VP neurons (***C***), cortical neurons (***D***), hippocampal CA1 neurons (***E***), ARC neurons (***F***), and neurons from other brain areas (Extended Data Fig. 2-1). Insets in ***A***, ***B*** show magnified areas (3 × 3 μm) outlined by small red squares on the corresponding images.

Previous work has shown that the subcortical layer of actin filaments found in isolated magnocellular neurons is essential for their activation in response to acute hypertonic stimuli (Zhang et al., 2007). To study whether this actin structure is also present in neurons *in situ*, we examined the organization of actin cytoskeleton by immunostaining rat brain tissue sections (Fig. 1; Extended Data Fig. 1-1). Consistent with previous studies in acutely isolated SON neurons (Zhang and Bourque, 2008), we revealed the presence of a subcortical layer of actin in magnocellular VP neurons from the rat SON *in situ* (Figs. 1*A*, 2*A*). Similar to SON magnocellular neurons, we found actin outlining MNC somata in the magnocellular division of the PVN (Fig. 1*B*), as well as in the accessory nuclei harboring magnocellular VP neurons (Extended Data Fig. 1-1*A*). Notably, similar subcortical actin organization was found in several additional brain areas, including hippocampal neurons (Fig. 1*E*; Extended Data Fig. 1-1*C*,*D*) and neurons in the hypothalamic arcuate nucleus (ARC; Fig. 1*D*), but was absent in neurons from other brain areas, for example, cortical neurons and VP neurons from the suprachiasmatic nucleus (SCN; Fig. 1*C*,*F*).

10.1523/ENEURO.0351-19.2020.f2-1 Extended Data Figure 2-1 Comparison of actin networks in neurons from different brain areas. Immunostaining for β-actin (white) and VP (red, ***A***, ***B***) or neuronal marker NeuN (red, ***C***, ***D***) in adult rat brain sections imaged by confocal microscopy with AiryScan in the magnocellular VP neuron from the accessory nucleus (***A***), VP neuron from the parvocellular division of the PVN (***B***), and hippocampal CA3 (***C***), and DG (***D***). Download Figure 2-1, TIF file.

**Figure 3.**
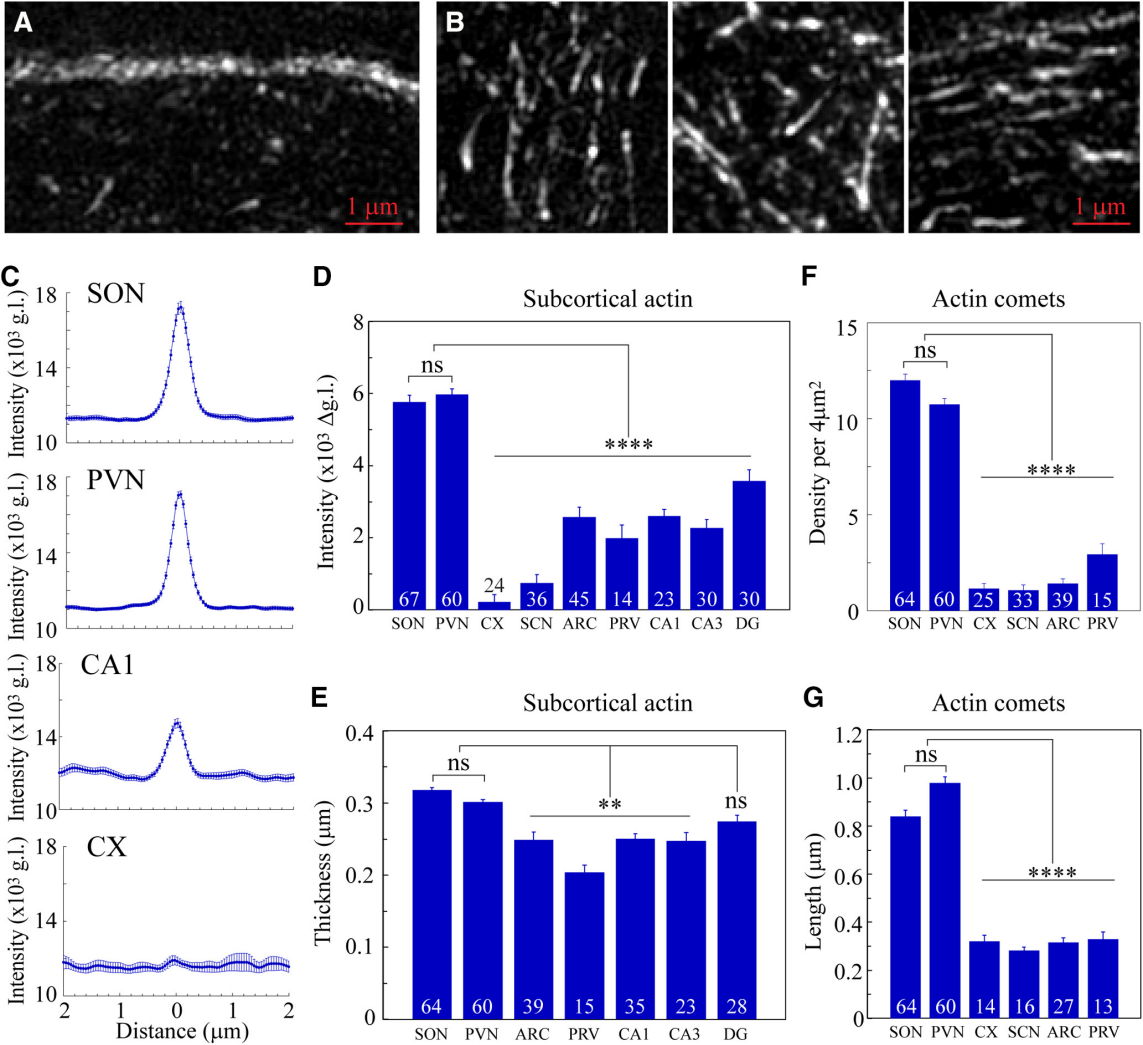
Analysis of actin networks in magnocellular neurons. Immunostaining for β-actin in adult rat brain sections imaged by super-resolution SIM reveals subcortical (***A***) and cytoplasmic comet-like (***B***) actin networks in magnocellular VP neurons. Sections through multiple brain areas of seven rats were examined for the analysis and quantification of the subcortical actin layer and cytoplasmic comet-like structures. ***C***, Line scan plots showing mean ± SEM values of actin fluorescence in g.l. as a function of distance from the cell perimeter, in magnocellular VP neurons from SON (67) and PVN (60), CA1 hippocampal neurons (30), cortical neurons (CX, 24); also see Extended Data Figure 3-1 for additional neuronal cell types. ***D***, Mean subcortical actin layer intensity in neurons from SON (67), PVN (60), CX (24), SCN (36), ARC (45), PVN parvocellular VP neurons (PRV, 14), and hippocampal CA1 (23), CA3 (30), and DG (30) neurons. ***E***, Mean subcortical actin layer thickness in neurons from SON (64), PVN (60), ARC (39), PRV (15), and hippocampal CA1 (35), CA3 (23), and DG (28) neurons. ***F***, Mean density of comet-like actin structures in magnocellular VP neurons from SON (64), PVN (60), CX (25), SCN (33), ARC (39), and PRV (15). ***G***, Mean length of comet-like actin structures in magnocellular VP neurons from SON (64), PVN (60), CX (14), SCN (16), ARC (27), and PRV (13). Data are presented as mean ± SEM; **p < 0.01, ****p < 0.0001; ns, not significant. Additional statistical parameters are shown in Extended Data Figure 3-2.

To gain more details on the organization of actin in neurons from different brain areas, we examined actin organization using confocal imaging with AiryScan (Fig. 2; Extended Data Fig. 2-1) and super-resolution with SIM (Fig. 3*A*,*B*). We analyzed the organization and density of actin in several populations of hypothalamic neurons expressing VP, comparing VP MNCs in the SON to VP neurons from the SCN, and VP neurons from both magnocellular and parvocellular divisions of the PVN, as well as other brain areas (Figs. 2, 3; Extended Data Fig. 2-1).

10.1523/ENEURO.0351-19.2020.f3-1 Extended Data Figure 3-1 Analysis of the subcortical actin network. Line scan plots showing mean ± SEM values of actin fluorescence as a function of distance from the cell perimeter in VP SCN neurons (36), ARC neurons (45), PVN parvocellular VP neurons (PRV, 14), and hippocampal CA3 (23) and DG (31) neurons. Download Figure 3-1, TIF file.

10.1523/ENEURO.0351-19.2020.f3-2 Extended Data Figure 3-2 Statistical analyses characterizing parameters of actin networks in neurons from different brain areas. Download Figure 3-2, XLSX file.

High-resolution analysis of rat brain tissue revealed the presence of a ∼0.3-μm-thick subcortical layer of actin filaments in magnocellular VP neurons from the rat SON *in situ* (Figs. 2*A*, 3*A*). In addition, a similar actin structure was found in the magnocellular portion of the PVN (Fig. 2*B*), as well as in magnocellular neurons from the accessory nucleus (Extended Data Fig. 2-1*A*). The intensity of this actin layer is higher in magnocellular SON and PVN VP neurons as compared with other types of VP neurons as well as cortical, hippocampal, and hypothalamic neurons (Fig. 2; Extended Data Fig. 2-1), as quantified by line scan and mean intensity analyses of subcortical actin immunofluorescence in different brain areas (Fig. 3*C*,*D*; Extended Data Fig. 3-1). Importantly, subcortical actin intensity appears to be similar in magnocellular VP neurons from the SON and PVN [SON 5762 ± 188 vs PVN 5977 ± 156 gray levels (g.l.), *p* = 0.995], but is significantly lower in neurons from other brain areas [cortex 224 ± 207, SCN 752 ± 229, ARC 2578 ± 270, parvocellular PVN 1985 ± 379, and hippocampal CA1 2608 ± 185, CA3 2280 ± 224, and dentate gyrus (DG) 3581 ± 304 g.l.; *p* < 0.0001; Fig. 3*D*]. These data suggest that magnocellular neurons exhibit a denser subcortical actin network compared with other neurons. Furthermore, while the thickness of the subcortical actin layer is similar in magnocellular VP neurons from the SON and PVN (SON 0.32 ± <0.01 vs PVN 0.30 ± <0.01 μm, *p* > 0.99), this subcortical layer appears to be missing in some areas (e.g., SCN and cortical neurons; Fig. 2*C*,*F*, respectively), and significantly thinner in neurons from most other brain areas: ARC 0.25 ± 0.01 *p* < 0.01, parvocellular PVN 0.20 ± 0.01 *p* < 0.0001, hippocampal CA1 0.25 ± 0.01 *p* < 0.01, and CA3 0.25 ± <0.01 *p* < 0.01, apart from DG 0.27 ± <0.01 μm *p* = 0.82; Figs. 2, 3; Extended Data Fig. 2-1).

In addition to the subcortical actin layer, we detected other actin structures in the somata of magnocellular VP neurons from the SON, PVN, and accessory nucleus. These actin filaments appear as a sparse array of short fibers distributed throughout the entire volume of the perinuclear cytoplasm (Figs. 2*A*,*B*, 3*B*; Extended Data Fig. 2-1*A*). These structures resemble comet-like filaments of up to 1 μm in length (Fig. 3*B*). The organization of these comet-like structures appears to be similar in magnocellular VP neurons of the SON and PVN, their cytoplasmic density (SON 12.0 ± 0.31 vs PVN 10.75 ± 0.30 filaments/4 μm^2^, *p* = 0.733; Fig. 3*F*) and length (SON 0.84 ± 0.03 vs PVN 0.98 ± 0.03 μm, *p* = 0.093; Fig. 3*G*) are not significantly different between magnocellular neurons of the SON and PVN. Importantly, these comet-like actin filaments are not found in any other type of neuron investigated (Figs. 2*C–F*, 3*F*,*G*; Extended Data Fig. 2-1*B–D*). Notably, these comet-like actin filaments do not resemble typical actin structures found in other cell types, such as stress fibers (Tojkander et al., 2012), or filopodia and lamellipodium structures found in a variety of motile cells (Lehtimäki et al., 2017). Thus, these comet-like structures represent a unique cytoskeletal network featured by magnocellular neurons.

### Salt-loading (SL) increases actin density in magnocellular VP neurons

**Figure 4.**
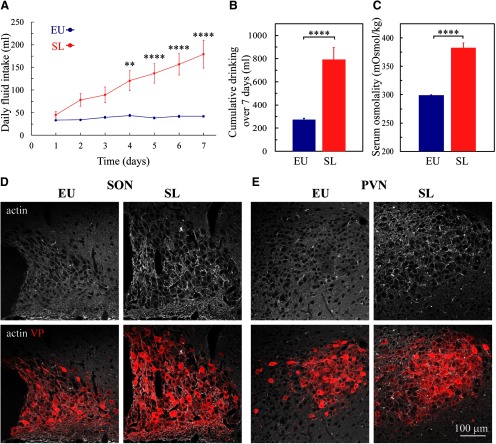
The effect of SL on actin in the SON and PVN. ***A***, Mean ± SEM values of daily fluid intake in rats subjected to SL by replacing their drinking solution with 2% NaCl compared with control EU rats drinking tap water *ad libitum*. ***B***, Mean ± SEM values of total fluid intake during 7 d in eight SL and eight EU rats. ***C***, Mean blood osmolality following 7 d of SL (eight) and EU rats (eight). Confocal images of immunostaining for β-actin (white) and VP (red) in brain sections of SON (***D***) and PVN (***E***) of EU and SL rats. Data are presented as mean ± SEM; **p < 0.01, ****p < 0.0001; ns, not significant.

To test whether chronic exposure of rats to elevated levels of dietary salt leads to an increase in the density of actin cytoskeleton in magnocellular VP neurons, we exploited a well-characterized model of high dietary salt-induced hypertension referred to as SL (Jones and Pickering, 1969; Velasquez and Alexander, 1982; Ludwig et al., 1996; Li et al., 1998; Osborn and Hornfeldt, 1998; Fujio et al., 2006; Choe et al., 2015). Rats exposed to 7 d of SL by replacing the drinking solution with 2% NaCl, develop hypernatremia (Li et al., 1998), increased plasma osmolality (Choe et al., 2015), and become progressively hypertensive (Choe et al., 2015). Consistent with these observations, we found that rats exposed to 7 d of SL gradually increase their fluid intake (Fig. 4*A*,*B*) and develop elevated plasma osmolality as compared with control rats drinking tap water *ad libitum* (Fig. 4*C*).

**Figure 5.**
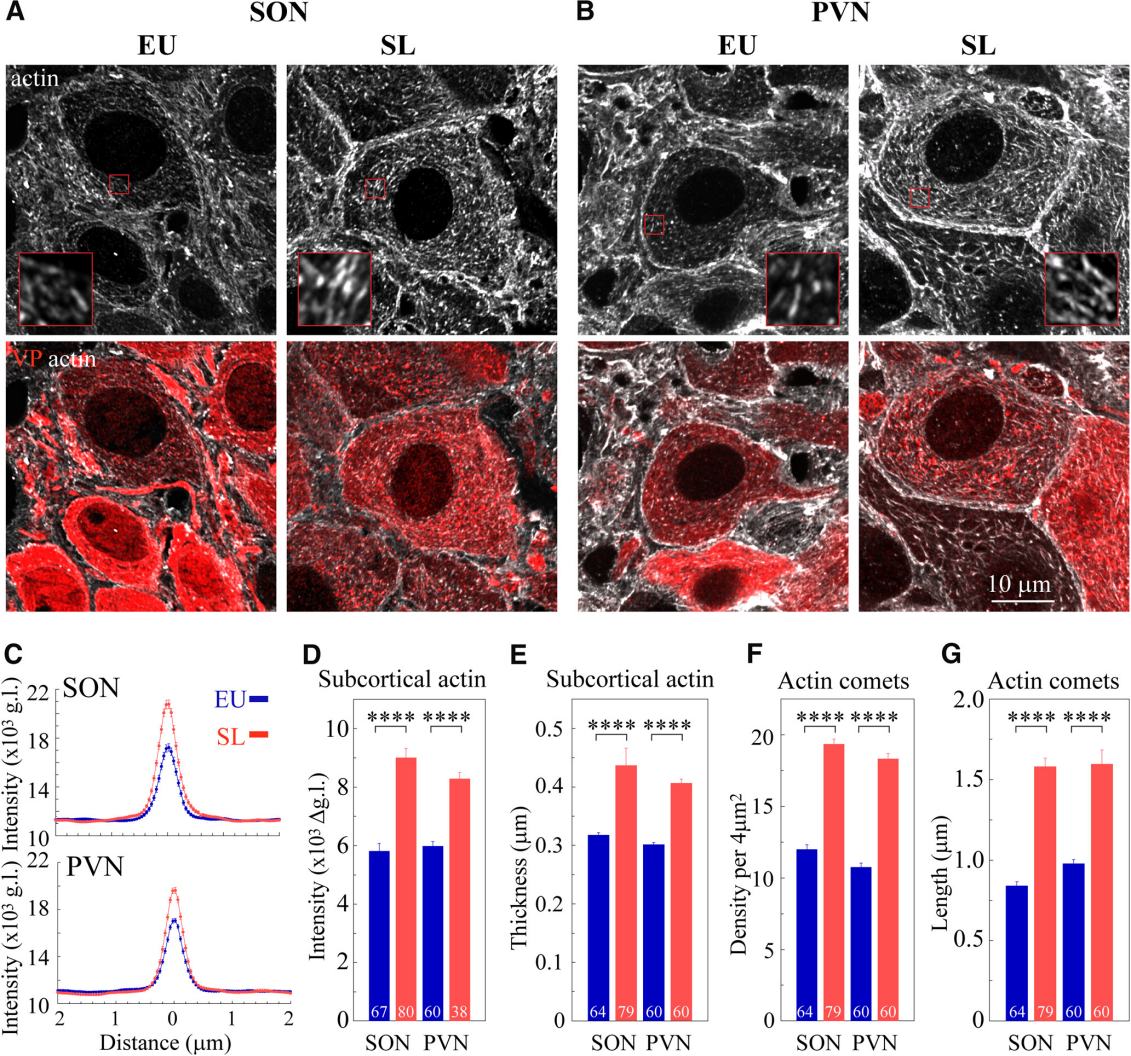
The effect of SL on subcortical and cytoplasmic actin networks in magnocellular VP neurons. Immunostaining for β-actin (white) and VP (red) in brain sections showing magnocellular VP SON (***A***) and PVN (***B***) neurons from a control (EU) rat and a rat subjected to 7 d of SL, imaged using confocal microscopy with AiryScan. Insets in ***A***, ***B*** show magnified areas (3 × 3 μm) outlined by small red squares on the corresponding images. The organization of subcortical and cytoplasmic actin networks in SON neurons from EU and SL was verified using two additional anti β-actin antibodies, as shown in Extended Data Figure 5-1. ***C***, Line scan plots showing mean ± SEM values of actin fluorescence as a function of distance from the cell perimeter, in magnocellular VP neurons from the SON VP neurons (upper plot: EU blue, 67 cells from seven rats; and SL red, 80 cells from eight rats); and PVN (lower plot: EU blue, 60 cells from seven rats and SL red, 38 cells from eight rats). ***D***, Mean subcortical actin layer intensity for VP magnocellular neurons from seven EU and eight SL rats. EU: 67 SON and 60 PVN cells, SL: 80 SON and 38 PVN cells. ***E***, Mean subcortical actin layer thickness for VP magnocellular neurons from seven EU and eight SL rats. EU: 64 SON and 60 PVN cells, SL 79 SON and 60 PVN cells. Mean density (***F***) and mean length (***G***) of comet-like actin structures for VP magnocellular neurons from seven EU and eight SL rats. EU: 64 SON and 60 PVN cells, SL: 79 SON and 60 PVN cells. Data are presented as mean ± SEM; ****p < 0.0001. Additional statistical parameters are shown in Extended Data Figure 5-2.

Next, we examined the density of actin cytoskeleton in magnocellular SON and PVN VP neurons from SL and control rats (Figs. 4*D*,*E*, 5). Examination of brain sections from control rats and rats exposed to 7 d of SL shows that actin levels are elevated in the SON and PVN from SL rats (Fig. 4*D*,*E*). Super-resolution imaging analysis of actin organization in magnocellular neurons from the SON and PVN reveals augmented subcortical and cytoplasmic actin networks in the somata of SON and PVN VP neurons following SL (Fig. 5*A*,*B*). Line scan analysis of actin immunofluorescence indicated that the intensity of the subcortical actin layer is increased in magnocellular VP neurons from SL rats as compared with control, EU animals (SON: EU 5762 ± 188 vs SL 8999 ± 317 g.l., *p* < 0.0001; PVN: EU 5977 ± 156 vs SL 8280 ± 222 g.l., *p* < 0.0001; Fig. 5*C*,*D*). In addition, the subcortical actin layer is significantly thicker in VP magnocellular neurons following SL (SON: EU 0.32 ± <0.01vs SL 0.44 ± 0.03, *p* < 0.0001; PVN: EU 0.30 ± <0.01 vs SL 0.41 ± <0.01 μm, *p* < 0.0001; Fig. 5*E*). Furthermore, the density of comet-like actin filaments in VP magnocellular neurons from the SON and PVN of SL rats is significantly elevated compared with EU controls (SON: EU 12.0 ± 0.31 vs SL 19.35 ± 0.33 filaments/4 μm^2^, *p* < 0.0001; and PVN: EU 10.75 ± 0.30 vs SL 18.32 ± 0.36 filaments/4 μm^2^ *p* < 0.0001; Fig. 5*F*). Likewise, these structures appear to be longer in magnocellular VP neurons from SL rats compared with EU rats (SON: EU 0.84 ± 0.03 vs SL 1.58 ± 0.05 μm, *p* < 0.0001; PVN EU 0.98 ± 0.03 vs SL 1.60 ± 0.09 μm, *p* < 0.0001; Fig. 5*G*).

10.1523/ENEURO.0351-19.2020.f5-1 Extended Data Figure 5-1 The effect of SL on subcortical and cytoplasmic actin networks in magnocellular VP neurons. Immunostaining using an alternative mouse monoclonal antibody (***A***) or rabbit monoclonal antibody (***B***) against β-actin (white) and VP (red) in brain sections showing magnocellular VP SON neurons from a control (EU) rat and a rat subjected to 7 d of SL, imaged using confocal microscopy with AiryScan. Download Figure 5-1, TIF file.

10.1523/ENEURO.0351-19.2020.f5-2 Extended Data Figure 5-2 Statistical analyses characterizing parameters of actin networks in SON and PVN neurons from control and SL rats. Download Figure 5-2, XLSX file.

### SL does not affect actin organization in non-osmosensory neurons

**Figure 6.**
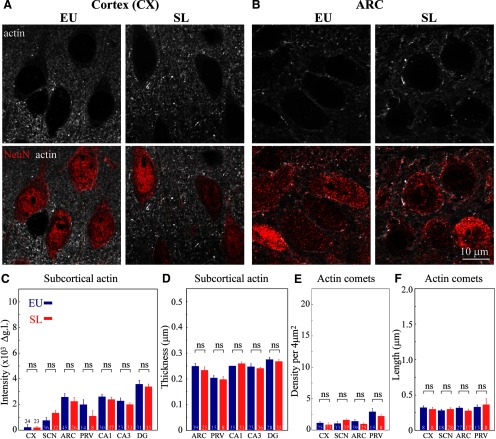
The effect of SL on actin networks in different brain areas. Immunostaining for β-actin (white) and NeuN (red) in brain sections showing cortical (CX, ***A***) and ARC (***B***) neurons from control (EU) and SL rats imaged using confocal microscopy with AiryScan (for additional brain areas, see Extended Data Figs. 6-1, 6-2, 6-3). Subcortical and cytoplasmic actin networks were analyzed in seven control (EU, blue) and eight SL (red) rats. ***C***, Mean subcortical actin layer intensity for different neuronal types, CX: 24 EU and 23 SL cells, VP SCN neurons: 36 EU and 32 SL cells, ARC: 45 EU and 26 SL cells, PVN parvocellular VP neurons: (PRV) 14 EU and eight SL cells, hippocampal CA1: 30 EU and 37 SL cells, CA3: 23 EU and 33 SL cells, and DG: 31 EU and 35 SL cells. ***D***, Mean subcortical actin layer thickness for different neuronal types, ARC: 39 EU and 25 SL cells, PVN parvocellular VP neurons: (PRV) 15 EU and eight SL cells, hippocampal CA1: 35 EU and 31 SL cells, CA3: 23 EU and 36 SL cells, and DG: 28 EU and 35 SL cells. ***E***, Mean density of comet-like actin structures for different neuronal cell types https://dx.doi.org/10.1523/ENEURO.0351-19.2020.f6-3, 3CX: 25 EU and 23 SL cells, VP SCN neurons: 33 EU and 37 SL cells, ARC: 39 EU and 25 SL cells, PVN parvocellular VP neurons: (PRV) 15 EU and eight SL cells. ***F***, Mean length of comet-like actin structures for different neuronal cell types, CX: eight EU and eight SL cells, VP SCN neurons: 18 EU and 28 SL cells, ARC: 27 EU and 17 SL cells, PVN parvocellular VP neurons: (PRV) 13 EU and eight SL cells. Data are presented as mean ± SEM; ns, not significant. Additional statistical parameters are shown in Extended Data Figure 6-4.

To examine whether the organization of actin is affected exclusively in magnocellular VP neurons, we investigated the organization and density of actin in other areas of the rat brain following 7 d of SL (Extended Data Figs. 6-1, 6-2, 6-3). We analyzed neurons from the cortex (Fig. 6*A*) and hippocampus (CA1, CA3, and DG areas; Extended Data Fig. 6-2*A–C*) and found that neurons in these areas do not exhibit changes in the organization of actin. In addition, we also examined the organization of actin in several additional hypothalamic areas, including ARC (Fig. 6*B*), SCN (Extended Data Fig. 6-2*D*), and the parvocellular division of the PVN (Extended Data Fig. 6-3*A*). Our data show that neurons in these areas do not display any changes in the thickness or density of subcortical actin following SL (Fig. 6*C*,*D*) as evaluated by the line scan analysis of actin immunofluorescence intensity in each brain area (Extended Data Fig. 6-3*C*). We also did not observe any changes in the density or size of comet-like actin structures in the analyzed brain areas (Fig. 6*E*,*F*).

10.1523/ENEURO.0351-19.2020.f6-1 Extended Data Figure 6-1 The effect of SL on actin in different brain areas. Confocal micrographs of immunostaining for β-actin (white) and neuronal marker NeuN (red in ***A***, ***B***, ***D–F***) or VP (red in ***C***, ***G***, ***H***) in brain sections from control (EU) and SL rats showing cortex (***A***), ARC (***B***), SCN (***C***), hippocampal CA1 (***D***), CA3 (***E***), and DG (***F***), parvocellular division of the PVN (***G***), and accessory nucleus (***H***). Download Figure 6-1, TIF file.

10.1523/ENEURO.0351-19.2020.f6-2 Extended Data Figure 6-2 The effect of SL on actin organization in different brain areas. Immunostaining for β-actin (white) and NeuN (red, ***A–C***) or VP (red, ***D***) in brain sections showing hippocampal neurons from CA1 (***A***), CA3 (***B***), and DG (***C***), and VP neurons from the SCN (***D***) in rat subjected to SL and control (EU), imaged using confocal microscopy with AiryScan. Download Figure 6-2, TIF file.

10.1523/ENEURO.0351-19.2020.f6-3 Extended Data Figure 6-3 The effect of SL on actin networks in different brain areas. Immunostaining for β-actin (white) and VP (red) in brain sections showing parvocellular VP neurons from the PVN (***A***) and magnocellular VP neurons from the accessory nucleus (***B***), in rat subjected to SL and control (EU), imaged by confocal microscopy with AiryScan. ***C***, Line scan plots showing mean ± SEM values of actin fluorescence as a function of distance from the cell perimeter, in cortical neurons (CX, 24 EU and 23 SL cells), VP SCN neurons (36 EU and 32 SL cells), ARC neurons (45 EU and 26 SL cells), PVN parvocellular VP neurons (PRV, 14 EU and eight SL cells), and hippocampal CA1 (30 EU and 37 SL cells), CA3 (23 EU and 33 SL cells), and DG (31 EU and 35 SL cells) from total seven control and eight SL rats. Download Figure 6-3, TIF file.

10.1523/ENEURO.0351-19.2020.f6-4 Extended Data Figure 6-4 Statistical analyses characterizing parameters of actin networks in neurons from different brain areas in control and SL rats. Download Figure 6-4, XLSX file.

Notably, similar to magnocellular neurons from the SON and PVN, we found that SL causes an enhancement of the subcortical and cytoplasmic actin networks in magnocellular VP neurons in the accessory nucleus (Extended Data Fig. 6-3*B*). These data further support the hypothesis that SL leads to the reorganization of the actin network specifically in magnocellular neurons, while neurons in other brain areas are not affected.

## Discussion

Previous studies suggest that MNCs are intrinsically osmosensitive and display cell autonomous responses to hypertonicity (Prager-Khoutorsky and Bourque, 2015; Prager-Khoutorsky, 2017). These intrinsic responses are mediated by the N-terminal variant of the TRPV1 channel (ΔN-TRPV1; Zaelzer et al., 2015) and require intact actin and microtubule networks (Prager-Khoutorsky et al., 2014). A recent study demonstrated that magnocellular neurons display a unique microtubule network that is not present in other neuronal cell types, which is essential for the intrinsic activation of VP neurons in response to hypertonicity (Prager-Khoutorsky et al., 2014). While it has been shown that actin is also a crucial component of this intrinsic machinery (Zhang et al., 2007; Zhang and Bourque, 2008; Prager-Khoutorsky and Bourque, 2010), the detailed organization of actin cytoskeleton in magnocellular neurons has not been investigated. In the present study, we aimed to characterize the organization of actin cytoskeleton in magnocellular neurons *in situ*, and to examine whether, similar to microtubules, actin forms a unique network in these neurons that differs from other neuronal types.

Using super-resolution imaging with SIM and AiryScan confocal microscopy analysis, we revealed that MNCs of both SON and PVN feature unique actin networks comprising a dense subcortical layer and a cytoplasmic array of comet-like actin structures *in situ*. Interestingly, the subcortical actin layer does not appear to be unique to magnocellular neurons and is found in additional neuronal cell types, e.g., hippocampal CA1, CA3, and DG neurons, as well as hypothalamic ARC and parvocellular PVN neurons. The size and density of this submembrane actin layer are variable across neuronal populations and are completely indistinguishable in some types of neurons (e.g., cortical and SCN neurons). However, the thickness and the density of this actin layer were found to be the greatest in magnocellular neurons (Figs. 2, 3). This does not appear to be related to the large size of MNCs, since some neuronal types which have comparable soma size (e.g., hippocampal CA1 and CA3 neurons), contain significantly smaller subcortical actin layer (Fig. 3*D*,*F*).

This subcortical layer in MNCs was proposed to be essential for creating a scaffold underneath the plasma membrane to provide mechanical support during hypertonicity-induced cell shrinkage and to transmit forces essential for the mechanical activation of the transduction channels (ΔN-TRPV1; Prager-Khoutorsky, 2017). The exact mechanism by which actin regulates ΔN-TRPV1 activation remains unclear, as previous studies have shown that actin does not bind directly to TRPV1 channels (Goswami et al., 2004; Goswami and Hucho, 2008), and thus the effect of the actin network on the activity of the transduction channel is likely to be indirect. Our results show that SL leads to an increase in the density of the subcortical actin layer in magnocellular VP neurons of the SON and PVN (Fig. 5), while actin cytoskeleton in other brain areas remains unchanged (Fig. 6). Previous studies have suggested that pharmacologically-induced increases in the density of actin enhances the cell autonomous responses of MNCs to osmolality (Zhang et al., 2007). This suggests that SL-induced modification of actin cytoskeleton in magnocellular VP neurons can lead to sensitization of this intrinsic osmosensitivity, leading to the increase in osmoregulatory gain (a relation between VP concentration and plasma osmolality; Prager-Khoutorsky and Bourque, 2010), to optimize fluid homeostasis in conditions associated with elevated plasma osmolality. This increase in the osmoregulatory gain mediating augmented activation of magnocellular VP neurons and enhanced VP release can be an adaptive mechanism that would be beneficial when blood osmolality is elevated due to dehydration and thus is crucial for the survival of the organism in this condition. While in conditions associated with chronic high-salt diet and hypernatremia, such as SL, increases in osmoregulatory gain may have pathologic consequences leading to enhanced fluid retention and vasoconstriction, thereby contributing to hypertension.

The molecular machinery that underlies the organization of the subcortical actin network in magnocellular neurons and mediates the modification of actin cytoskeleton in response to SL remains unknown. Previous studies suggest that angiotensin II can enhance intrinsic osmosensitivity of MNCs via its effect on the actin cytoskeleton (Zhang and Bourque, 2008; Prager-Khoutorsky and Bourque, 2010). Angiotensin II is involved in the regulation of blood pressure and fluid homeostasis via activation of the Rho family of small GTPases (Balakumar and Jagadeesh, 2014). Rho GTPases such as Rac and RhoA are recognized as master regulators of actin cytoskeleton that can mediate increases in actin filament density via two major classes of actin-biding proteins, Arp2/3 (Rotty et al., 2013) and formins (Breitsprecher and Goode, 2013), respectively. Thus, the angiotensin II/Rho pathway represents an attractive candidate that may control the reorganization of the subcortical actin layer in MNCs in conditions associated with high dietary salt and hypernatremia. Additional studies will be required to investigate the possible involvement of this pathway in the regulation of MNC activity.

Furthermore, we identified a novel cytoskeletal structure that appears to be a unique feature of magnocellular neurons. These actin structures appear as comet-tail-like filaments of ∼1 μm in length, sparsely distributed throughout the MNC somata (Figs. 2, 3*B*). These comet-like structures were not found in neurons from any other brain areas, including cortex, hippocampus, and hypothalamic SCN and ARC. Moreover, these somatic comet-like actin filaments have never been described in adult neurons *in situ* or *in vitro*. Interestingly, recent studies identified several actin structures present in axonal compartments of cultured neurons, including actin rings, hot spots, and trails (Leterrier et al., 2017; Papandréou and Leterrier, 2018). These structures are associated with different actin-binding proteins and vary in their dynamic properties. For example, actin rings are periodic submembrane structures spaced by spectrin and containing the capping protein adducin, and these rings appear to be very stable and immobile (Xu et al., 2013). Whereas actin trails, which arise from endosome-associated actin hot spots, undergo continuous polymerization and depolymerization, and are dependent on formins (Ganguly et al., 2015). Since actin rings, hot spots, and trails were characterized in axons of cultured neurons, it is unclear if they are also present in neuronal soma or found *in situ* or *in vivo.* In addition to neuronal axons, a recent study showed centrosome-associated actin puncta in early-developing pre-mitotic neurons in culture (Meka et al., 2019). As adult differentiated neurons lack centrosomes and microtubule organization in post-mitotic neurons depends on non-centrosomal nucleation (Sánchez-Huertas et al., 2016), it is unlikely that these actin structures can exist in mature neurons, since they were shown to depend on the presence of intact centrosome (Meka et al., 2019).

Notably, although actin comet-like structures in MNCs do not resemble typical actin organization in non-neuronal cell types (Svitkina, 2018), such as stress fibers found in fibroblasts, epithelial and endothelial cells (Tojkander et al., 2012) or Arp2/3-dependent actin meshwork found in a variety of motile cells (Lehtimäki et al., 2017), they resemble filamentous actin structures propelling organelles such as endosomes and endocytic vesicles (Collins et al., 2011; Svitkina, 2018) and bacteria comet tails (Tilney and Portnoy, 1989; Cameron et al., 2000, 2001; Svitkina, 2013). These structures are thought to be involved in organelle/vesicle trafficking and feature fast polymerizing actin filaments nucleated by Arp2/3 (Collins et al., 2011) or formins (Borinskaya et al., 2016). Future studies are needed to study dynamic properties of comet-like structures found in MNC somata and characterize their interacting partners, to determine whether these comet-like filaments resemble axonal actin trails, organelle-propelling actin filaments, or represent a functionally distinct structure in MNCs that might be crucial for their unique physiology. While the functional role of MNC actin comets remains to be explored, it is conceivable that these structures are involved in the trafficking of VP secretory vesicles, and conditions associated with an increased demand for hormonal release, such as SL (Dunn et al., 1973; Ludwig et al., 1996), require facilitated VP transport and upregulation of VP trafficking machinery to support this massive secretion.

In conclusion, our findings reveal a unique actin network found exclusively in magnocellular neurons and show that this network is modified in response to chronic exposure to high dietary salt. This modification may contribute to the hyperactivation of the neurons and exaggerated VP secretion, constituting a new potential mechanism contributing to salt-sensitive hypertension.

## Synthesis

Reviewing Editor: Quentin Pittman, University of Calgary Decisions are customarily a result of the Reviewing Editor and the peer reviewers coming together and discussing their recommendations until a consensus is reached. When revisions are invited, a fact-based synthesis statement explaining their decision and outlining what is needed to prepare a revision will be listed below. The following reviewer(s) agreed to reveal their identity: Deepak Nair. Note: If this manuscript was transferred from JNeurosci and a decision was made to accept the manuscript without peer review, a brief statement to this effect will instead be what is listed below.

The reviewers and I agree that the paper titled “Unique organization of actin cytoskeleton in magnocellular vasopressin neurons in normal conditions and in response to salt-loading” is an interesting study that highlights comet-like actin organization in MNCs and that this organization changes in conditions following exposure to high dietary salt. Furthermore, the manuscript has improved considerably in data analysis and statistics from the previous version as well as in the clarity of presentation and making more of a connect between introduction and discussion. However, some of the major concerns still remain which has not been addressed by the authors.

1.The main message of the paper is a novel organization of actin. A major point of the previous review was the request to show that a different antibody labeled these structures. No doubt you have argued persuasively that the antibody is well validated (something that should be indicated in your methods). However, the worry is not the specificity of the antibody but the structures not stained by phalloidin. Some of these structures seems to be more than the critical length for filamentous form but not stained for f-actin as you point out (you attribute it to cofilin). That is the reason to request the experiment with a second antibody to show that these structures are indeed detected by another beta actin. There are two possibilities:

a) This structure is specific to this clone of antibody (AC-15). It could be an artifact of labeling with the specific clone. Thus, the need for a second antibody.

b) The F-actin does not label the actin comets due to changes in conformation or masking by another protein like Cofilin which the reviewers have suggested.

It does not seem unreasonable to request that in control tissue you provide an example of use of another actin antibody to see if the comet labeling can be reproduced/verified by a different Beta-actin antibody or that you show that comets are labeled with other proteins like cofilin which is the key to the interpretation of this paper. It is certainly not necessary to do so in all figures.

2. Your statistics are much improved. However, there are things that remain unclear. You used the normality test to ascertain the quality of the distribution, but also indicated that there are some distributions that are not normal. If not normal, you need to report median and IQR but not mean +/- SEM. Considering there have mixed populations it would be ideal to report both. (Editor note: you may wish to consider estimation statistics as suggested in my letter to you)

Additional suggestions for revision:

4. Literature Survey: Some of the papers that have been cited by the authors, especially the work of Ganguly et al and literature from other systems like Tillney and Portnoy, 1989 and other works from Leek and Liu, Biophys J, 2008 Cameron et al Nature Rev. Mol. Cell Bio 200 all talk about comet like structures which in some cases they refer to as “actin trails”. From the images it does not look any different through the Ganguly et al has evaluated this only in Axons. Furthermore the same lab which published Ganguly et al has published another manuscript (Meka at al, EMBO reports, 2019) in developing neurons that shows similar structures for actin. It would be interesting to the community to discuss the “actin comets” in light of these previous observations. There is still a feeling that lines 395-397 is a bit of stretch in the discussion.

5. As understood from the results there is not enough data to support the claims in a discussion from lines 357-362 and 381-390. It more or looks like a farfetched extrapolation of the literature review in the introduction.

6.The authenticity of methods: Most of the experiments that are being used here use airy scan which gives a resolution around 120-130 nm and SIM around 120 nm for 1 (SIM-only for image 3 (a,b)). These are more like resolution doubling than state of the art super-resolution experiments which perform below 50nm. Please clarify why you have used SIM since the abbe scan and SIM almost gives the same axial and lateral resolutions.

Minor Comments

1) Figure 2: It would be great if you could show an arrow to the structures the illustrate the comets. And also provide an inset of some of the structures at the scale of 1-3 micrometers

2) What is the relationship between images in Figures 3A and B? Is it only representational or is it from the same cell? error bars are not visible on some of the panels in Figure 3C.

3) Figure 4D, are these images for quantification or for representative purpose.?What is the reader expected to notice in this image? Figure 4A blue line-scale bars not very visible

4) Figure 5. It would be great to provide a scale bar for the grayscales that is being presented in the top panel Figure 5A EU and SL conditions. Please provide insets too at higher zoom.

5) Figure 5C. Error bars not very visible. In figure 5 E the red and blue curves indicating the thickness. Here red seems to be significantly larger than blue.
