# Supplementary figures and images for: Unique Organization of Actin Cytoskeleton in Magnocellular Vasopressin Neurons in Normal Conditions and in Response to Salt-Loading
Source: eNeuro. 2020 Apr 7;7(2):ENEURO.0351-19.2020. doi: 10.1523/ENEURO.0351-19.2020 (PMC7189486; doi:10.1523/ENEURO.0351-19.2020)

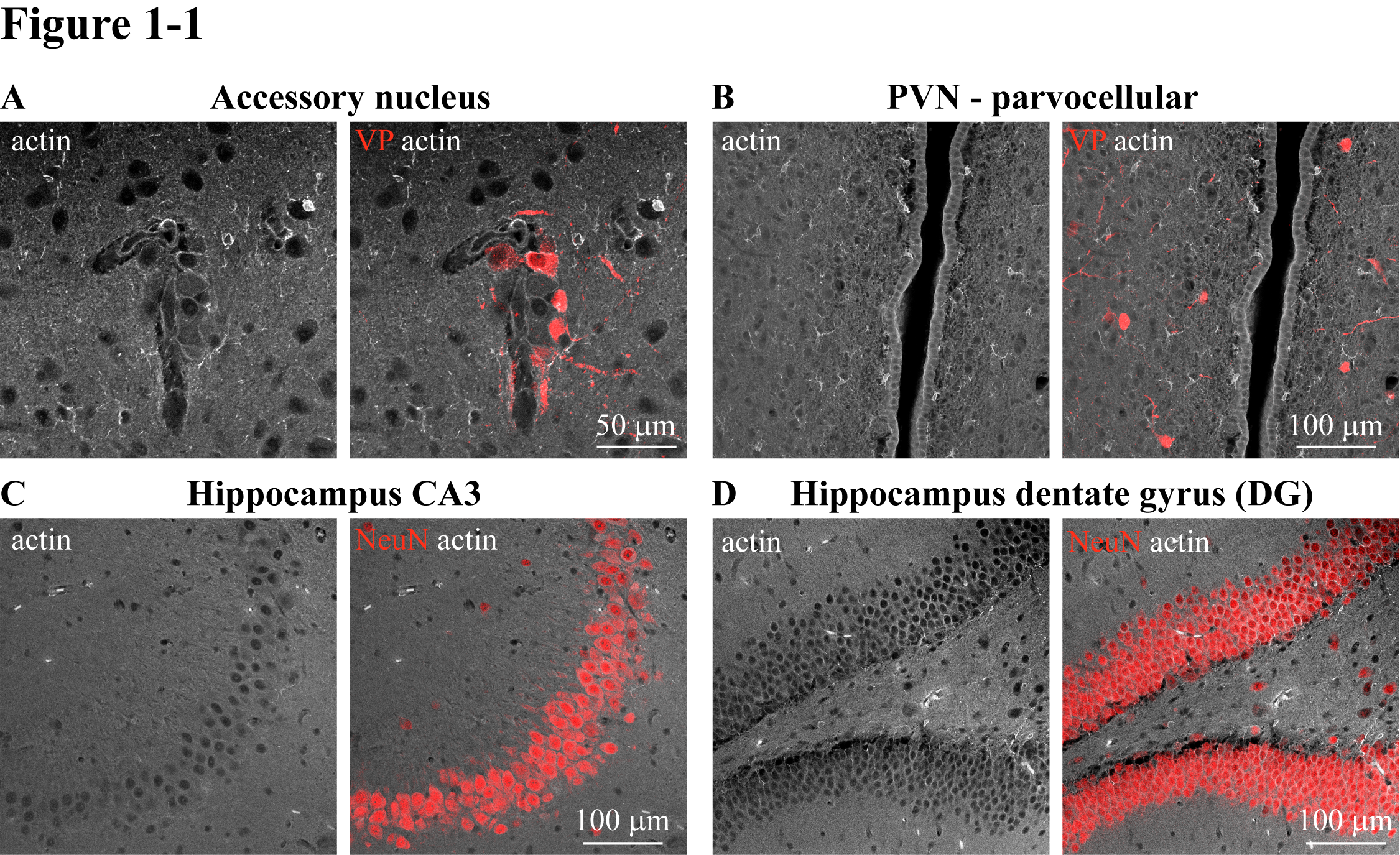

Supplement: Extended Data Figure 1-1 — Examination of actin organization in different brain areas. Confocal imaging of immunostaining for β-actin (white) and VP (red, A, B) or neuronal marker NeuN (red, C, D) analyzed in adult rat brain sections containing accessory nucleus harboring magnocellular VP neurons (A), parvocellular division of the PVN (B), and hippocampal CA3 (C) and DG (D). Download Figure 1-1, TIF file. [file enu-eN-NWR-0351-19-s02.tif]

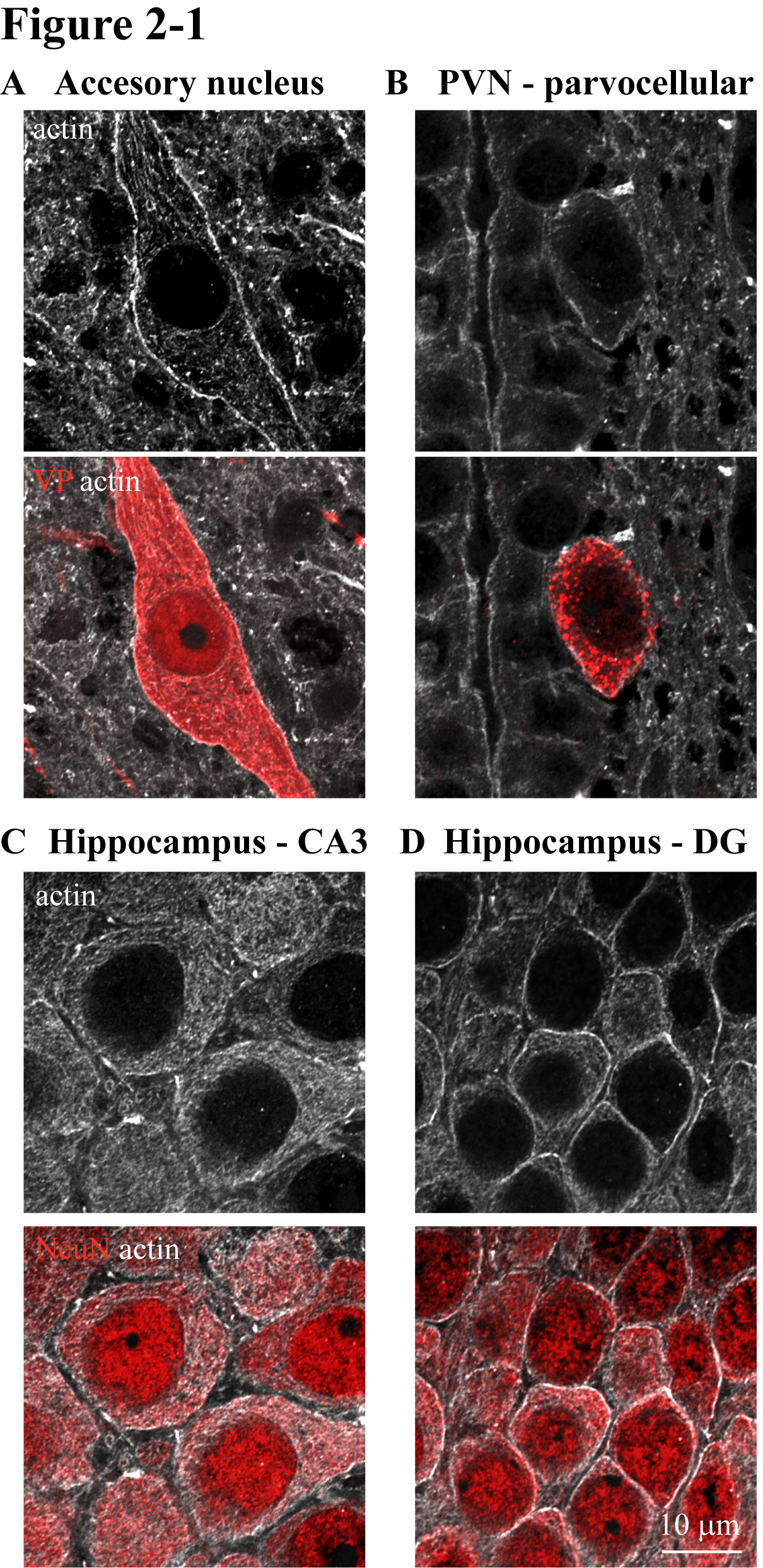

Supplement: Extended Data Figure 2-1 — Comparison of actin networks in neurons from different brain areas. Immunostaining for β-actin (white) and VP (red, A, B) or neuronal marker NeuN (red, C, D) in adult rat brain sections imaged by confocal microscopy with AiryScan in the magnocellular VP neuron from the accessory nucleus (A), VP neuron from the parvocellular division of the PVN (B), and hippocampal CA3 (C), and DG (D). Download Figure 2-1, TIF file. [file enu-eN-NWR-0351-19-s03.tif]

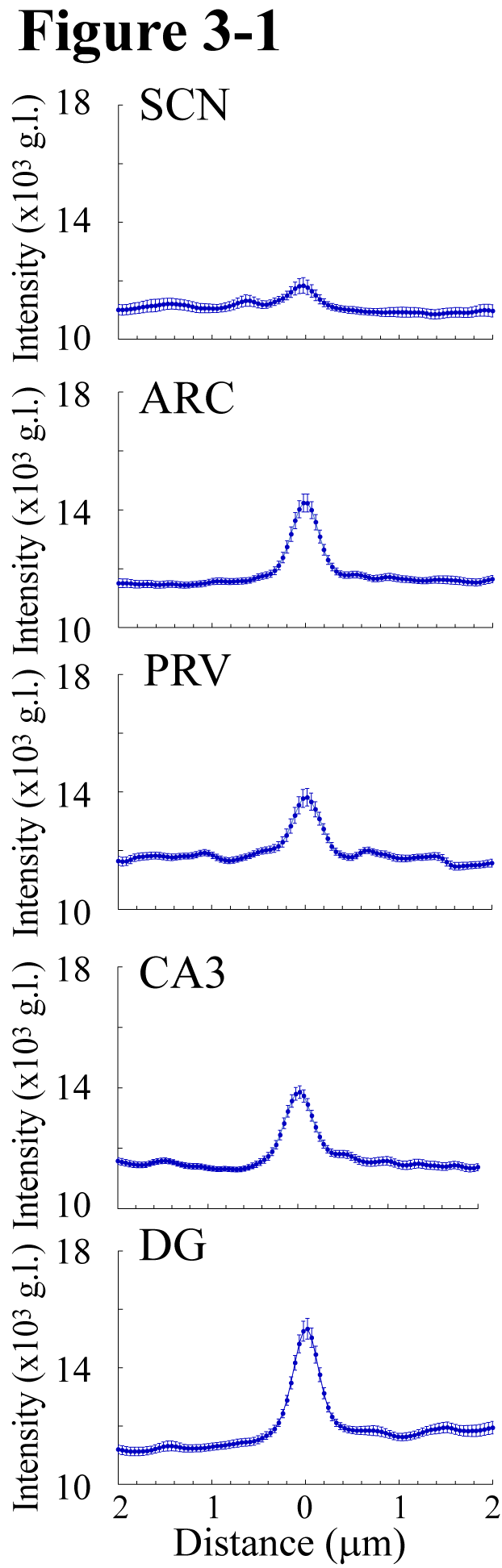

Supplement: Extended Data Figure 3-1 — Analysis of the subcortical actin network. Line scan plots showing mean ± SEM values of actin fluorescence as a function of distance from the cell perimeter in VP SCN neurons (36), ARC neurons (45), PVN parvocellular VP neurons (PRV, 14), and hippocampal CA3 (23) and DG (31) neurons. Download Figure 3-1, TIF file. [file enu-eN-NWR-0351-19-s04.tif]

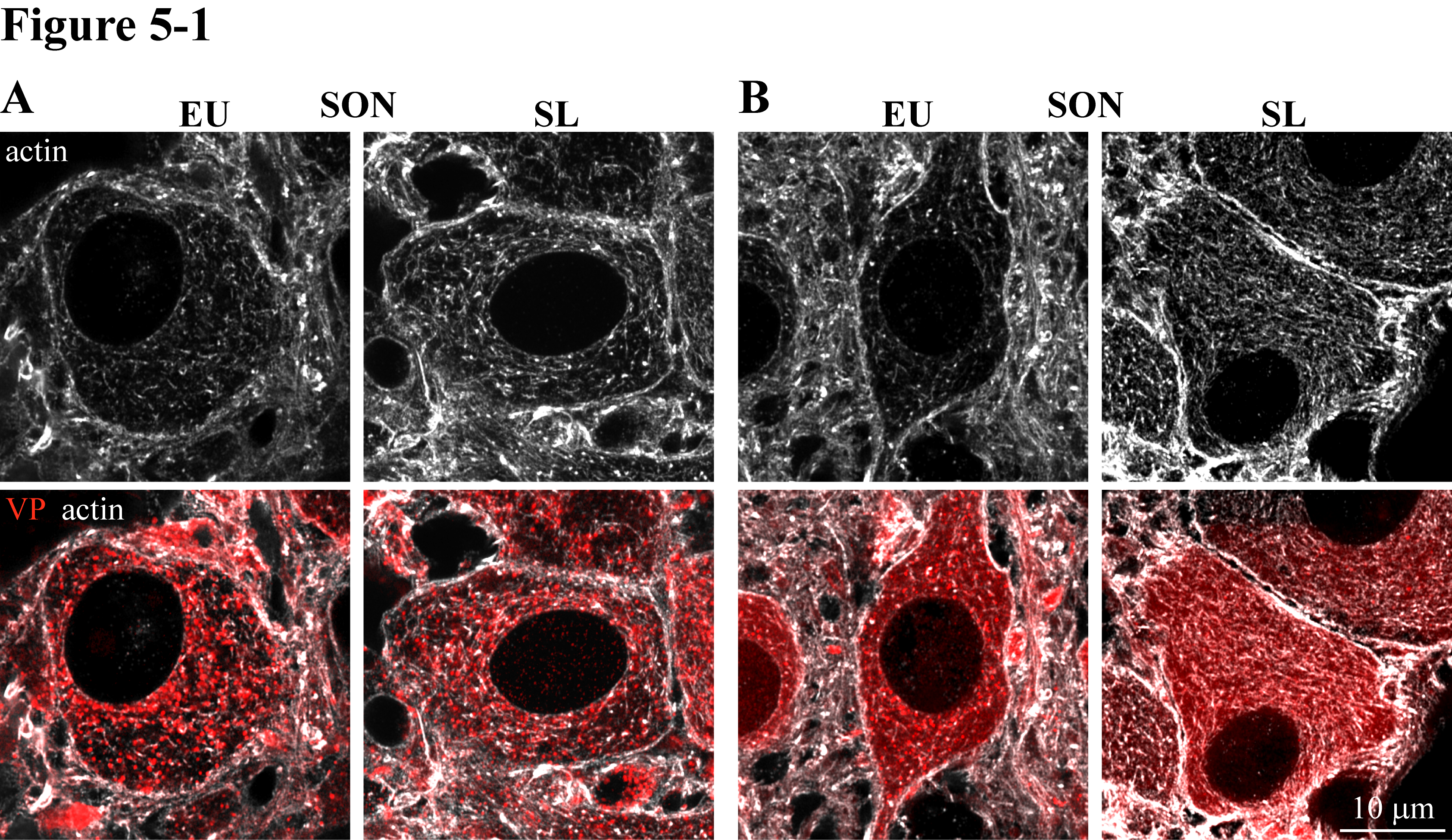

Supplement: Extended Data Figure 5-1 — The effect of SL on subcortical and cytoplasmic actin networks in magnocellular VP neurons. Immunostaining using an alternative mouse monoclonal antibody (A) or rabbit monoclonal antibody (B) against β-actin (white) and VP (red) in brain sections showing magnocellular VP SON neurons from a control (EU) rat and a rat subjected to 7 d of SL, imaged using confocal microscopy with AiryScan. Download Figure 5-1, TIF file. [file enu-eN-NWR-0351-19-s06.tif]

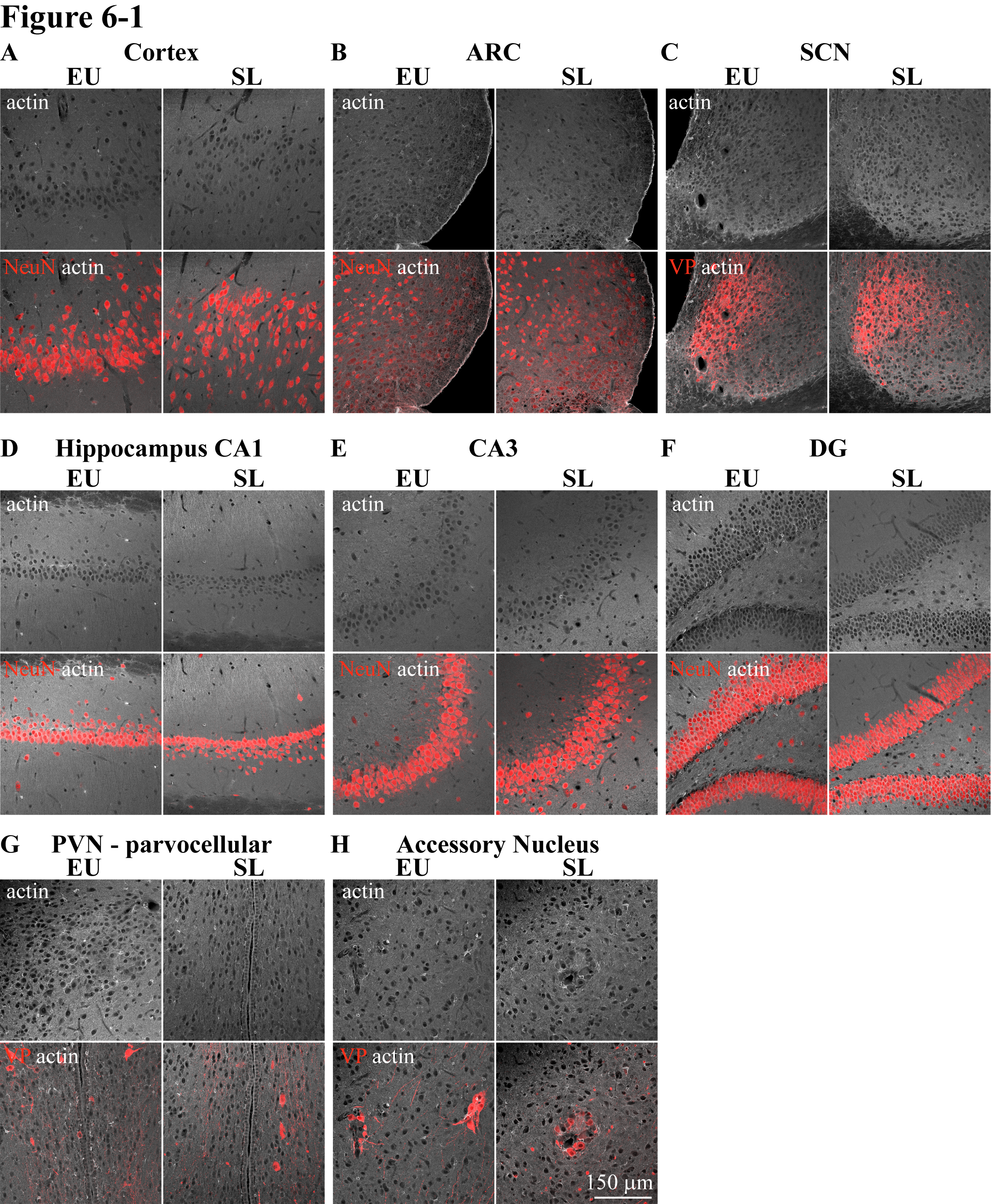

Supplement: Extended Data Figure 6-1 — The effect of SL on actin in different brain areas. Confocal micrographs of immunostaining for β-actin (white) and neuronal marker NeuN (red in A, B, D–F) or VP (red in C, G, H) in brain sections from control (EU) and SL rats showing cortex (A), ARC (B), SCN (C), hippocampal CA1 (D), CA3 (E), and DG (F), parvocellular division of the PVN (G), and accessory nucleus (H). Download Figure 6-1, TIF file. [file enu-eN-NWR-0351-19-s08.tif]

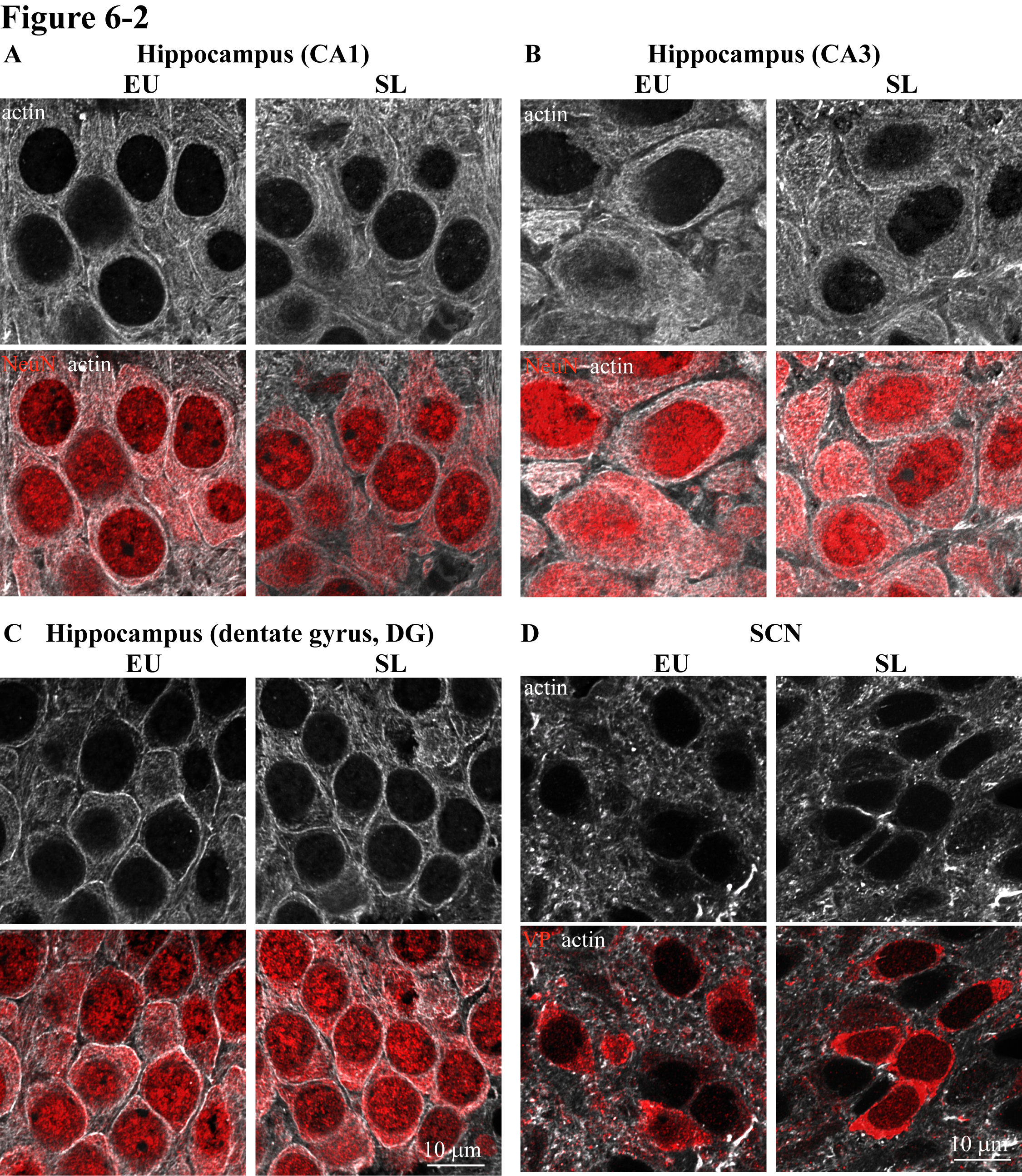

Supplement: Extended Data Figure 6-2 — The effect of SL on actin organization in different brain areas. Immunostaining for β-actin (white) and NeuN (red, A–C) or VP (red, D) in brain sections showing hippocampal neurons from CA1 (A), CA3 (B), and DG (C), and VP neurons from the SCN (D) in rat subjected to SL and control (EU), imaged using confocal microscopy with AiryScan. Download Figure 6-2, TIF file. [file enu-eN-NWR-0351-19-s09.tif]

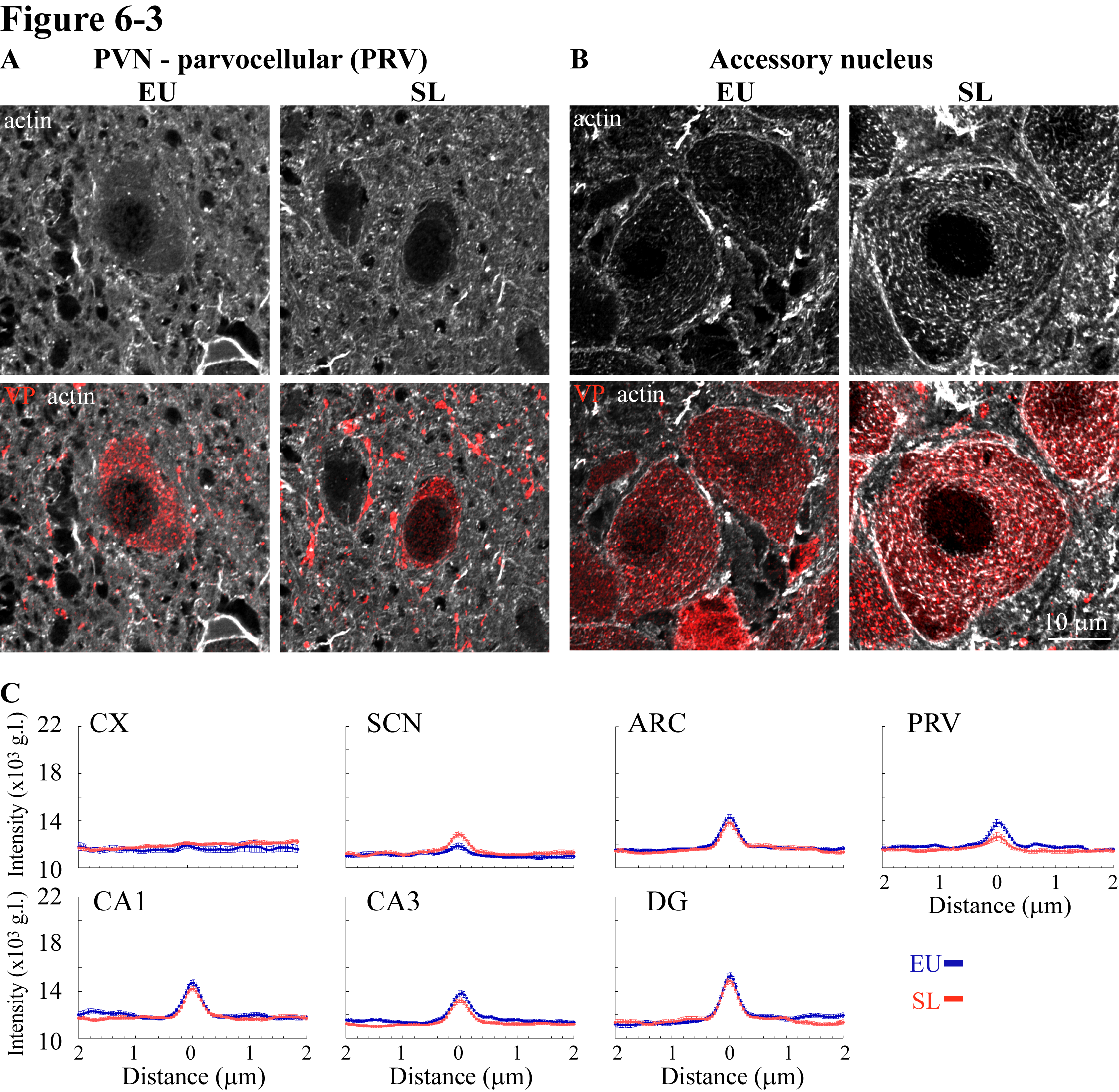

Supplement: Extended Data Figure 6-3 — The effect of SL on actin networks in different brain areas. Immunostaining for β-actin (white) and VP (red) in brain sections showing parvocellular VP neurons from the PVN (A) and magnocellular VP neurons from the accessory nucleus (B), in rat subjected to SL and control (EU), imaged by confocal microscopy with AiryScan. C, Line scan plots showing mean ± SEM values of actin fluorescence as a function of distance from the cell perimeter, in cortical neurons (CX, 24 EU and 23 SL cells), VP SCN neurons (36 EU and 32 SL cells), ARC neurons (45 EU and 26 SL cells), PVN parvocellular VP neurons (PRV, 14 EU and eight SL cells), and hippocampal CA1 (30 EU and 37 SL cells), CA3 (23 EU and 33 SL cells), and DG (31 EU and 35 SL cells) from total seven control and eight SL rats. Download Figure 6-3, TIF file. [file enu-eN-NWR-0351-19-s10.tif]
